# Comparative transcriptome analysis reveals a regulatory network of microRNA-29b during mouse early embryonic development

**DOI:** 10.18632/oncotarget.10741

**Published:** 2016-07-20

**Authors:** Ying Wang, Tao Zhou, Jinyuan Wan, Ye Yang, Xiaojiao Chen, Jiayi Wang, Cheng Zhou, Mingxi Liu, Xiufeng Ling, Junqiang Zhang

**Affiliations:** ^1^ Department of Reproduction, Nanjing Maternity and Child Health Care Hospital, Affiliated to Nanjing Medical University, Nanjing 210004, P.R. China; ^2^ State Key Laboratory of Reproductive Medicine, Department of Histology and Embryology, Nanjing Medical University, Nanjing 210029, P.R. China

**Keywords:** miR-29b, Nanog, Klf4, embryogenesis, reprogramming

## Abstract

MicroRNAs are endogenous ~22 nt RNAs that regulate gene expression by translational inhibition and mRNA destabilization. MicroRNA-29b (miR-29b) is essential for progression of mouse embryos past preimplantation development; however, details of the underlying regulatory network remain to be elucidated. Here, we used RNA sequencing to identify changes in the transcriptome of mouse embryos in response to miR-29b inhibition. Morula-stage embryos that had been subject to miR-29b inhibition throughout preimplantation development exhibited significant expression changes in 870 genes compared with controls. Among 405 genes that were downregulated, 30 genes encoded factors with known essential function during early embryonic development, including the pluripotent stem cell factor Nanog. We identified 19 genes encoding putative miR-29b target transcripts. These included *Zbtb40*, *Hbp1*, *Ccdc117*, *Ypel2*, *Klf4*, and *Tmed9*, which are upregulated at the 4-cell state of mouse development concomitant with miR-29b downregulation. Luciferase reporter analysis confirmed that *Zbtb40*, *Hbp1*, *Ccdc117*, *Ypel2*, and *Klf4* transcripts are direct targets of miR-29b. These results suggest that miR-29b decreases the mRNA levels of several target genes during early mouse development, including the gene encoding the reprogramming factor Klf4. We hypothesize that inhibition of miR-29b causes overexpression of its target genes, triggering downstream signaling networks to decrease the expression of genes that are essential for embryonic development. In conclusion, miR-29b controls an extensive regulatory network in early mouse embryos, which comprises reprogramming factors and molecular regulators of post-transcriptional modification processes.

## INTRODUCTION

MicroRNAs (miRNAs) are ~22 nucleotide (nt) noncoding RNAs that generally bind through imperfect base-pairing to the 3'untranslated regions (3' UTRs) of their target mRNAs to suppress the translation and stability of these mRNAs [1]. Although the regulatory networks and target genes of most miRNAs remain unclear, diverse miRNAs have been shown to participate in processes such as embryonic development, differentiation, organogenesis, growth, cell proliferation and apoptosis [2-8]. Studies in pluripotent stem cells suggest that multiple miRNA species regulate developmental processes in the early embryo. For example, miRNA species have been identified that are exclusive to embryonic stem cells and enhance the production of induced pluripotent stem cells (iPSCs) when introduced into somatic cells [9]. Other miRNAs, including miR-134, miR-145, miR-296 and miR-470 become upregulated during differentiation and have been found to inhibit pluripotency factors such as Oct4, Sox2 and Nanog [10]. These data strongly imply miRNAs in fate decisions in the early embryo.

It has previously been shown that Sox2 directly regulates endogenous miR-29b expression during iPSC generation and that miR-29b expression is required for OSKM (Oct4, Sox2, Klf4, and c-Myc) and OSK (Oct4, Sox2 and Klf4)-mediated reprogramming [11]. During preimplantation development, miR-29b is highly expressed at the 2-cell stage, concomitant with genomic activation, whereas 4-cell, 8-cell, morula and blastocyst stage embryos express only low levels [12]. Thus, miR-29b appears to play a role predominantly during early embryonic development, prior to the first cell fate decision in the embryo, which occurs during the 5- to 8-cell stage [13]. Inhibition of miR-29b during early embryogenesis causes developmental delay before the blastocyst stage without visible morphological changes at earlier preimplantation stages [12]. This developmental arrest may in part be due to disruption of DNA methylation-related reprogramming events in the early embryo, which are regulated by miR-29b: Alterations in miR-29b activity affect expression levels of DNMT (DNA (cytosine-5-)-methyltransferase), resulting in altered global methylation levels [12]. Thus, miR-29b plays a major role in the regulation of DNA methylation-related reprogramming events in the early embryo and iPSCs. The network of miR-29-regulated targeted genes in the early embryo remains has not been explored, and the identity of target genes linked to the developmental arrest of miR-29b depleted embryos remains unknown.

Here, we used RNA-seq to identify changes in the transcriptome of mouse preimplantation stage in response to miR-29b inhibition. Morula-stage embryos that had been subject to miR-29b inhibition throughout preimplantation development exhibited significant expression changes in 870 genes compared with controls. Our study validated differentially expressed genes and indicates that regulatory activity of miR-29b in the early embryo includes direct interaction with the 3' UTR of select target transcripts.

## RESULTS

### Inhibition of miR-29b alters the expression of 870 genes in mouse preimplantation stage embryos

**Figure 1:**
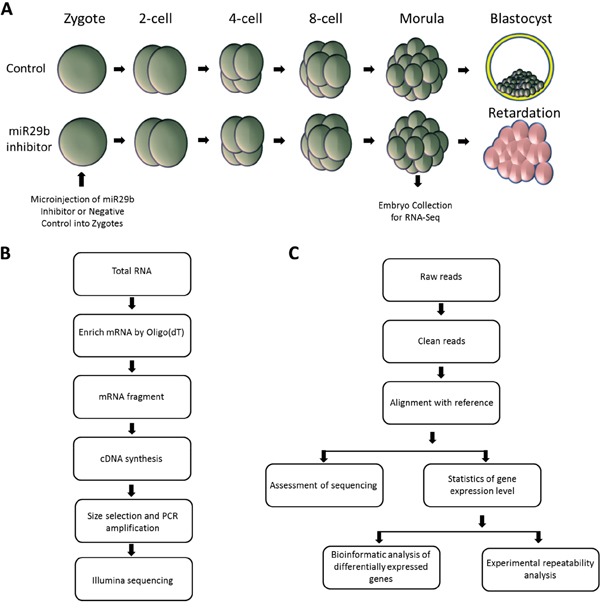
Experimental strategy for comparative transcriptome analysis **A.** Schematic depiction of preimplantation development following microinjection of miR29b inhibitor or vehicle control. Embryos were collected at the morula stage; **B.** cDNA library build and sequencing; **C.** Workflow of standard bioinformatics analysis and validation.

To suppress miR-29b activity during preimplantation development, we injected zygote stage embryos with a commercially miR-29b inhibitor. As observed previously [12], zygotes injected with miR-29b inhibitor or mock negative control developed into morphologically similar morula stage embryos when cultured in vitro. For global transcriptome analysis using RNA-seq, 10 morula stage embryos were collected per experimental group, which included miR-29b inhibitor-injected, mock negative control-injected, and untreated embryos (Figure 1A). After library preparation and sequencing (Figure 1B), RNA-Seq analysis was used to identify differentially expressed genes (DEGs) (Figure 1C). Assessment of sequencing data included quality assessment of the reads, sequencing saturation analysis, and randomness assessment (Supplementary Figures S1-S3). Over 98% of the clean reads from each group were collected and used for comparative analysis.

Transcript levels were quantified using normalized RPKM (reads per kilobase per million) (Supplementary Table S1). To verify that mock-injected negative controls were similar in status to normal embryos that had not been subject to microinjection, we also assessed the difference between the normal and negative control groups by sample clustering, which did not reveal any transcriptional differences between groups (Supplementary Figure S4). Following validation of the negative control, comparison of transcriptomes of the miR-29b inhibition group with the mock-injected negative control group yielded a total of 870 DEGs that met criteria for significance.

### DEGs include genes involved in metabolism, glycosylation, and development

**Figure 2:**
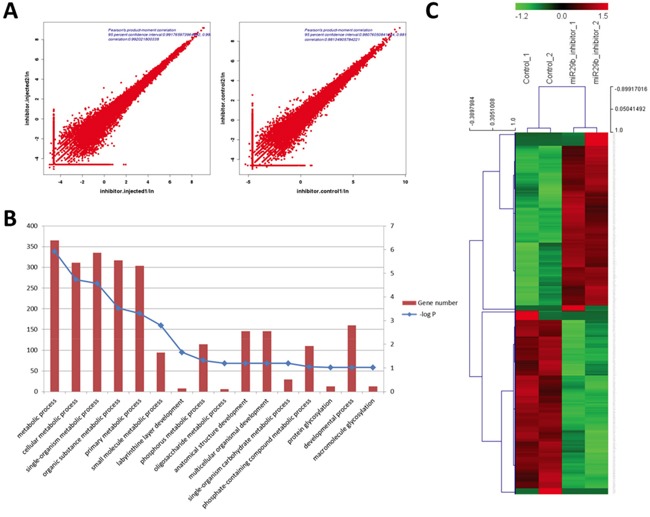
Identification of DEGs between morula-stage embryos subject to miR-29b inhibition and mock-injected controls **A.** Pearson's product-moment correlation of samples in each group; **B.** Biological process GO terms analysis of DEGs. Left and right Y-axes represent the number of DEGs and P-value of enrichment, respectively; **C.** Heat map cluster analysis of DEGs from each group.

Correlations across biological replicates were high in both the miR-29b inhibition group and in the control group (Pearson's product moment correlation=0.992 and 0.981; Figure 2A). The functional annotation of DEGs revealed enrichment of genes that are involved in various metabolic processes, protein glycosylation, and developmental processes (Figure 2B). The 870 DEGs included 465 upregulated DEGs (UDEGs) and 405 downregulated DEGs (DDEGs) (Figure 2C).

**Figure 3:**
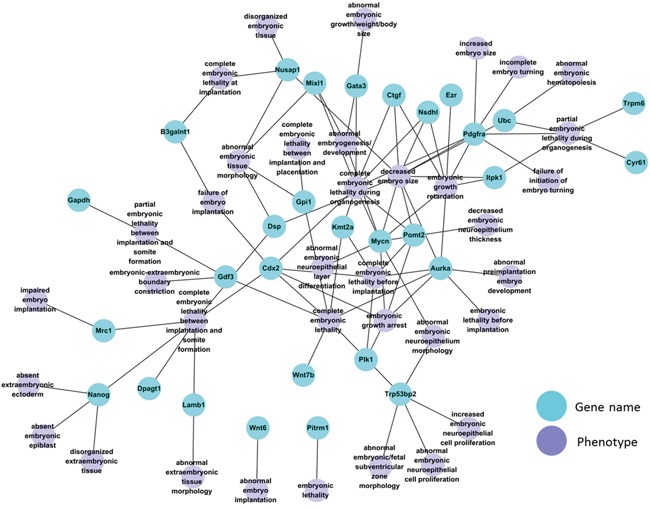
Network of 30 DDEGs with known embryonic phenotype in gene knockout mouse models

Among the DDEGs were 30 genes with essential function during early embryonic development according to Mouse Genome Informatics (MGI) database annotations, including the gene encoding the pluripotent stem cell factor Nanog (Figure 3). We used the DBTMEE database (http://dbtmee.hgc.jp/) [14] to accumulate transcriptome data sets that reflect expression levels of each of the 30 genes during early preimplantation development, specifically at the 1-cell, 2-cell and 4-cell stage. Heat map clustering of these patterns of the 25 genes for which data were available revealed that during normal embryonic development, expression levels of 6 DDEGs (*Pomt2*, *Nanog*, *Dpagt1*, *Mixl1*, *Cyr61*, and *Ctgf*; see Supplementary Table S2 for gene names) normally increase significantly between the 2- and the 4-cell stage (Supplementary Figure S5A; Supplementary Table S3). The products of these 6 genes are essential for mouse development (Table 1). Homozygous *Pomt2* knockout mice die before implantation and arrest at the morula stage [15]; mice homozygous for a disruption in *Dpagt1* exhibit peri-implantation lethality due to widespread cell death [16]; mice homozygous for a disruption in *Nanog* die between E3.5 and E5.5 with abnormal and excessive formation of embryonic tissue [17]; homozygous *Mixl1* null embryos arrest by day 9 of embryonic development (E9) with severe developmental abnormalities [18]; *Cyr61* null mice die around midgestation with failure in chorioallantoic fusion or placental vascular insufficiency and compromised vessel integrity [19]; and deficiency for *Ctgf* causes skeletal dysmorphisms as a result of impaired chondrocyte proliferation and disrupted extracellular matrix composition within the hypertrophic zone [20]. Upregulation of these 6 genes at the 4-cell stage (Supplementary Figure S5A) implies that the products of these genes may already play important roles in early stage preimplantation embryos. These expression patterns may be subject to transcriptional and or post-transcriptional regulation, and miR-29b may contribute to this regulation. We chose only one time point for our comparative transcriptome analysis of the early-stage embryos. Quantitative PCR-based analysis validated that *Pomt2*, *Nanog*, *Dpagt1*, *Mixl1*, *Cyr61*, and *Ctgf* transcripts were downregulated in morula-stage embryos response to miR-29b inhibition (Supplementary Figure S5C).

**Table 1:** Summary of DDEGs that are normally upregulated during early embryonic development

Gene Name	Symbol	Mouse null mutant phenotype	Function
Protein-O-mannosyltransferase 2	Pomt2	Preimplantation lethal, arrest at the morula stage	Glycosylation
Nanog homeobox	Nanog	Arrest E3.5-E5.5 with abnormal and excess embryonic tissue development	Transcription factor
Dolichyl phosphate (UDP-N-acetylglucosamine) acetylglucosamine phosphotransferase 1 (GlcNAc-1-P transferase)	Dpagt1	Peri-implantation lethality.	Glycosylation
Mix1 homeobox-like 1 (Xenopus laevis)	Mixl1	Mostly arrested by E9, abnormal morphology	Transcription factor
Cysteine rich protein 61	Cyr61	Arrest around E10.5, defects in chorioallantoic fusion	Extracellular matrix binding
Connective tissue growth factor	Ctgf	Perinatal lethal with respiratory failure	Fibronectin binding, growth factor activity

### Predicted targets of miR-29b include reprogramming factors

**Figure 4:**
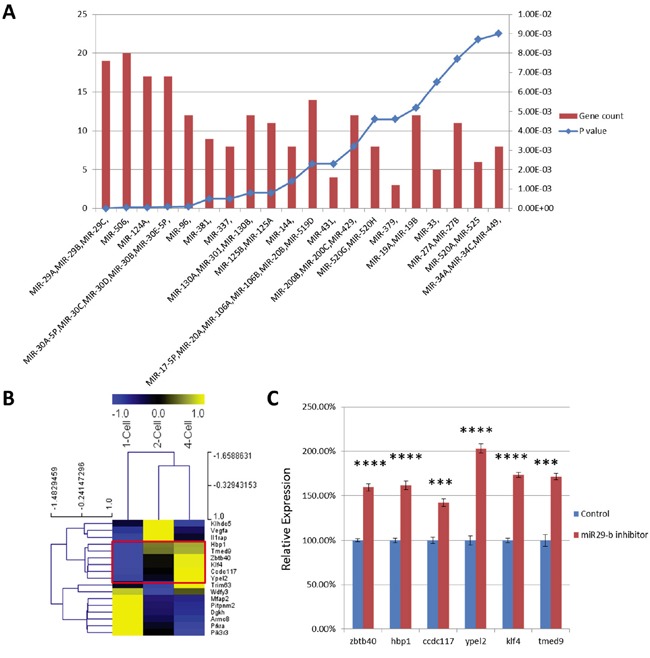
**A.** MicroRNA target analysis of UDEGs. Left and right Y-axes represent number of UDEGs and P-value of enrichment, respectively. **B.** Heat map cluster analysis of transcript levels of candidate genes in normal embryos at the 1-cell, 2-cell and 4-cell stage. Normalized FPKM values are represented from blue to yellow; **C.** Relative expression of 6 UDEGs in morula stage embryos that had been injected with miR-29b inhibitor (red) or mock control (blue) at the zygote stage. The 6 genes analyzed are normally upregulated during early preimplantation stages (marked by red box in B). ***, P-value < 10^-3^; ****, P-value < 10^-4^

To identify putative target genes of miR-29 in preimplantation stage embryos, we next performed micro-RNA target prediction analyses of the coding regions of UDEG transcripts. Confirming the validity of our experimental approach, UDEGs were highly enriched for predicted target genes of the miR-29 cluster (Figure 4A). We identified 19 candidate genes that were predicted targets of miR-29b (Table 2). Heat map cluster analysis of expression levels of these genes during early preimplantation development (DBTMEE database) showed that expression levels of *Zbtb40*, *Hbp1*, *Ccdc117*, *Ypel2*, *Klf4*, and *Tmed9* normally increase between the 2-cell stage and the 4-cell stage (Figure 4B), whereas miR-29b expression levels decrease at this stage of development [12]. One of these genes, *Klf4*, encodes a known important factor involved in pluripotent stem cell reprogramming [21-23]. Quantitative PCR analysis confirmed significant downregulation of transcripts levels of 6 predicted miR-29 target genes in morula-stage embryos after miR-29b inhibition (Figure 4C).

**Table 2:** Candidate miR-29b target genes with significant upregulation after miR-29b inhibition

Gene Symbol	GeneID	Fold change	P value
Zbtb40	230848	2.3159485	0.0083416
Prkra	23992	2.6428584	0.0295639
Dnm3	103967	2.5164698	0.0394192
Mafb	16658	3.7176978	0.0034471
Hbp1	73389	2.2868132	0.0175397
Wdfy3	72145	2.055516	0.047755
Trim63	433766	5.0926565	0.0039434
Vegfa	22339	NC	0.0102822
Dgkh	380921	NC	0.0102822
Il1rap	16180	10.864414	0.0109375
Pitpnm2	19679	2.5164698	0.0394192
Ccdc117	104479	2.7642867	0.0035426
Ypel2	77864	3.3816921	0.0105161
Mfap2	17150	NC	0.0016988
Klf4	16600	2.1484607	0.0498541
Tmed9	67511	3.4171257	0.0097273
Klhdc5	232539	3.2186964	0.0243587
Pik3r3	18710	2.767989	0.0295077
Armc8	74125	2.0020686	0.0084944

Fold change represents the normalized RPKM value of the miR-29b inhibition group relative to the control group; NC indicates that the transcript was not detectable in the control group.

### Zbtb40, Hbp1, Ccdc117, Ypel2 and Klf4 transcripts are direct targets of miR-29b

**Figure 5:**
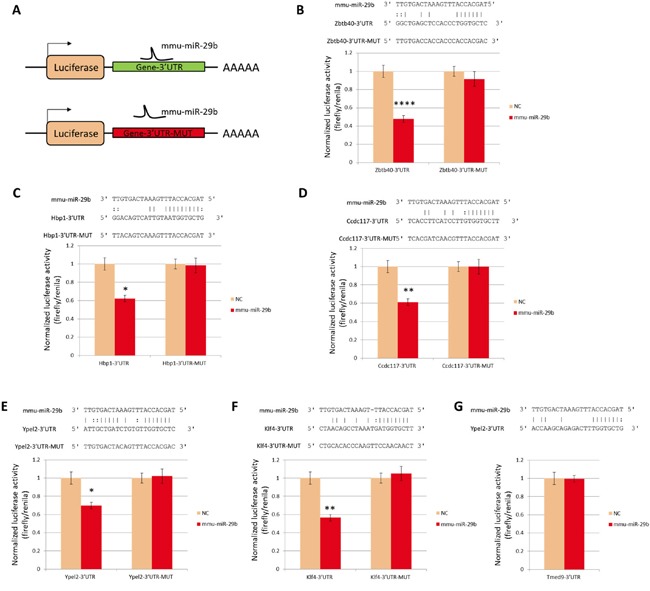
Luciferase reporter analysis of miR-29b targets **A.** Schematic drawing of reporter constructs. **B-G.** Normalized reporter activity at 48 hours after co-transfection of constructs with mmu-miR-29b mimic or mock negative control (NC). Sequences illustrate predicted base pairing between miR-29b and the 3'UTR of target genes, and absence of complementarity of miR-29b with mutant target sequences used as negative controls. **: P<0.01. NC, (B) Zbtb40, (C) Hbp1, (D) Ccdc117, (E) Ypel2, (F) Klf4 and (G) Tmed9;

To explore whether miR-29b may directly interact with any of the target candidate genes, we evaluated the 3'-untranslated region (3' UTR) sequences of candidate mRNAs and found that *Zbtb40*, *Hbp1*, *Ccdc117*, *Ypel2*, *Klf4*, and *Tmed9* transcripts contained predicted miR-29b target sequences according to the miRNA database (miRNA.org; http://www.microrna.org/) and RNA22 (http://omictools.com/rna22-tool) (Figure 5). To validate these interactions, we performed luciferase reporter assays with wild type or mutant 3' UTR sequences of candidate genes cells in 293T cells. Mmu-miR-29b mimics reduced luciferase expression from reporter constructs containing the 3' UTR sequences of *Zbtb40*, *Hbp1*, *Ccdc117*, *Ypel2*, or *Klf4* (Figure 5B-5F) but not *Tmed9* (Figure 5G) or any construct with 3'UTR sequences modified to abolish putative binding (Figure 5B-5F). These findings indicate that the 3' UTR sequences of *Zbtb40*, *Hbp1*, *Ccdc117*, *Ypel2*, and *Klf4* transcripts are direct targets of miR-29b.

## DISCUSSION

We have previously shown that inhibition of miR-29b during preimplantation development leads to early embryonic arrest [12]. This phenotype may be caused by the down-regulation of multiple genes. Here we have unraveled global changes in the transcriptome of early mouse embryos following disrupting miR-29b function in vivo. Three DDEGs—*Nanog* [17], *Mixl1* [18] and *Kmt2a* [24]—encode known factors that play unique roles in embryogenesis. These genes may not be direct targets of miR-29b, but they may still be key components of the miR-29b regulatory network.

Furthermore, 3 DDEGs—*Pomt2* [15], *Dpagt1* [16] and *B3galnt1* [25]—have previously been reported to be involved in glycosylation, which is essential for embryogenesis: In mouse models, disruption of these genes results in pre- or peri-implantation embryonic lethality. We identified 13 other DEGs associated with the glycosylation process. Outcomes of previous studies have indicated that miR-29b not only regulates protein glycosylation but also blocks the actions of the Plk1 and Aurka protein kinases thus promoting embryonic lethality before implantation [26, 27]. These results indicate that the miR-29b regulatory network affects various post-transcriptional modifications including glycosylation and phosphorylation.

The general function and biogenesis of micro-RNAs are increasingly well understood [28]. Additionally, it is possible to predict target transcripts of micro-RNAs by analyzing their sequence features. Among genes with expression changes in response to miR-29b inhibition in the early embryo, we identified 30 genes that were significantly decreased and associated with abnormal embryonic phenotypes reported in knockout mouse models. Differentially affected genes included the gene for the reprogramming factor Nanog, which is involved in transcriptional regulation and cell fate decisions during preimplantation development from the 8-cell stage to the blastocyst stage [13]. Interestingly, another reprogramming factor—Sox2—binds to the miR-29b promoter and stimulates miR-29b expression in iPSCs. Moreover, one of the miR-29b candidate targets—Klf4—is also a known reprogramming factor. These data suggest that miR-29b participates in early embryonic reprogramming events by regulating the expression of multiple reprogramming factors. Because we have identified only a few candidate miR-29b target genes using known sequence features predicting interactions with miRNAs, it is likely that many of the observed changes in the transcriptomes in response to miR-29b inhibition occur secondary to changes in direct target genes such as *Klf4*.

In summary, a comparative transcriptome analysis of the miR-29b inhibition group and the control group revealed the regulatory network of miR-29b, which comprises reprogramming factors and molecular regulators of the post-transcriptional modification processes that occur during mouse early embryonic development.

## MATERIALS AND METHODS

### Reagents and animals

All reagents for embryo in vitro culture were obtained from Sigma Chemicals (St. Louis, MO, USA) unless otherwise indicated. ICR mice were housed in a controlled environment of 20–22°C, 50–70% humidity, 12/12-h light/dark cycle, with ad libitum access to food and water. Animal care and all experimental procedures were in accordance with the Animal Research Committee guidelines of Nanjing Medical University (China).

### Embryo collection and culture

For zygote collection, 8-week-old female ICR mice were injected intraperitoneally (i.p.) with pregnant mare serum gonadotropin (PMSG, 10 IU) followed by i.p. injection of human chorionic gonadotropin (HCG, 10 IU) 48 h later. Females were mated with male ICR mice. After 15–17 h, zygotes were obtained from the oviducts and placed into Hepes-buffered CZB medium. Cumulus cells were dispersed with 1 mg/ml hyaluronidase in Hepes-CZB. Cumulus-free zygotes were washed with Hepes-CZB medium and then cultured in CZB medium at 37°C in a humidified atmosphere of 5% CO_2_.

### Microinjection of miR-29b inhibitor or mock control into zygotes

A commercially available mirVana^®^ miRNA Inhibitor (Applied Biosystems, California, US) specific for mmu-miR-29b (mouse miR-29b), was microinjected into the cytoplasm of zygotes as previously described [12, 29]. The mirVana^®^ miRNA Inhibitor Negative Control #1 (Applied Biosystems, 4464084) was used as the mock negative control. These reagents (approximately 5–7 pl per zygote) were microinjected at 100 nM, while non-injected zygotes served as the normal control groups. A Nikon Diaphot ECLIPSE TE 300 inverted microscope (Nikon, Yuko, Japan), equipped with Narishige MM0-202N hydraulic three-dimensional micromanipulators (Narishige Inc., Tokyo, Japan) was used for these experiments. After microinjection, the zygotes were washed and cultured in CZB medium for further observations of embryonic development. They were then collected for comparative transcriptome analysis.

### RNA-seq library preparation and sequencing analysis

Total RNA samples were treated with DNase I followed by enrichment of mRNA with oligo(dT) magnetic beads. Following fragmentation into strands of approximately 200 bp, first strand cDNA was synthesized using a random hexamer-primer reagent. Buffer, dNTPs, RNase H and DNA polymerase I were added to the mixture to synthesize the second strand. The double strand cDNA was purified using magnetic beads, followed by end repair and 3'-end single nucleotide A (adenine) addition. After ligation of sequencing adaptors, fragments were enriched by PCR amplification and the resulting sample libraries were subject to qualitative and quantitative QC using the Agilent 2100 Bioanalyzer and ABI StepOnePlus Real-Time PCR System.

The RNA-Seq analysis (IlluminaHiSeqTM 2000) was performed by the Beijing Genomics Institute (BGI) using an established protocol. The genome build used for the alignment was mm9. For alignment, clean reads were mapped to reference sequences and/or a reference gene set using SOAP2 [30]. No more than 2 mismatches were allowed in the alignment. Data were filtered by BGI as follows to obtain high quality reads: (1) removal of reads with adaptor sequences; (2) removal of reads in which the percentage of unknown bases (N) was greater than 10%; (3) removal of low quality reads. If the percentage of low quality bases (base with a quality value ≤ 5) was greater than 50% in a read, the read was defined as a low quality read.

### Statistical analysis and DEG identification

The values determined by the normalized Reads Per Kilobase of exon model per Million mapped reads (RPKM) for each gene were further adjusted by the mean value of each sample as described [31]. To identify differentially expressed genes (DEGs) between groups, we combined Student's t-test and a fold-change criterion: A P-value of less than 0.05 with a corresponding fold change greater than 2 (in the inhibitor injection group) were considered statistically significant. Hierarchical clustering analyses were performed based on the Pearson correlation and average linkage method using J-Express and MeV at both gene and sample levels [32, 33]. WebGestalt was used to identify enriched functional terms among the DEGs including regulatory microRNA, biological functions, pathways, and phenotypes [34, 35]. For microRNA target annotation, WebGestalt integrated microRNA binding sites that were inferred from the comparative genomic analysis and made available through MSigDB [36]. Fisher's exact test was applied to calculate the P value for enrichment. The mouse genome was set as the background and an adjusted P value less than 0.05 (using the Benjamini & Hochberg method) was defined as statistically significant.

### RNA extraction and quantitative RT-PCR

Total RNA from preimplantation stage embryos was extracted using the RNeasy MicroKit (Qiagen, 74034). Quantitative PCR analysis was carried out using SYBR^®^ Premix EX Taq (Takara, DRR420A) as described previously [37-39], and the 18S rRNA was used as the internal control. Sequences of primers used for quantitative PCR analysis are listed in Supplementary Table S4. Quantitative RT-PCR was performed using the ABI Step One sequence detection system (Applied Biosystems) using the following thermal cycling conditions: 30 sec at 95° C followed by 40 cycles of 5 sec at 95° C, 31 sec at 55° C, and 30 sec at 72° C. The 18S rRNA was amplified in parallel and used as the loading control.

### Luciferase reporter assay

Reporter constructs were generated by cloning cDNA sequences encoding Zbtb40-3'-UTR, Zbtb40-3'-UTR-MUT, Hbp1-3'-UTR, Hbp1-3'-UTR-MUT, Ccdc117-3'-UTR, Ccdc117-3'-UTR-MUT, Ypel2-3'-UTR, Ypel2-3'-UTR-MUT, Klf4-3'-UTR, Klf4-3'-UTR-MUT, or Tmed9-3'-UTR into the pGL3-3'UTR reporter plasmid (Promega). Plasmids were co-transfected into 293T cells with either miR-29b mimics or negative control. Firefly and Renilla luciferase activities were analyzed using the Dual-Luciferase Reporter Assay System according to the manufacturer's instructions (Promega) [40]. Relative luciferase activity was calculated by normalizing firefly luciferase activity to Renilla luciferase activity.

## SUPPLEMENTARY MATERIALS FIGURES AND TABLES




