# Supplementary material for: Comparative transcriptome analysis reveals a regulatory network of microRNA-29b during mouse early embryonic development
Source: Oncotarget. 2016 Jul 20;7(33):53772–82. doi: 10.18632/oncotarget.10741 (PMC5288220; doi:10.18632/oncotarget.10741)
Supplement: Supplementary file 1 [file oncotarget-07-53772-s001.pdf]

Comparative transcriptome analysis reveals a regulatory network of microRNA-29b during mouse early embryonic development

SUPPLEMENTARY FIGURES AND TABLES

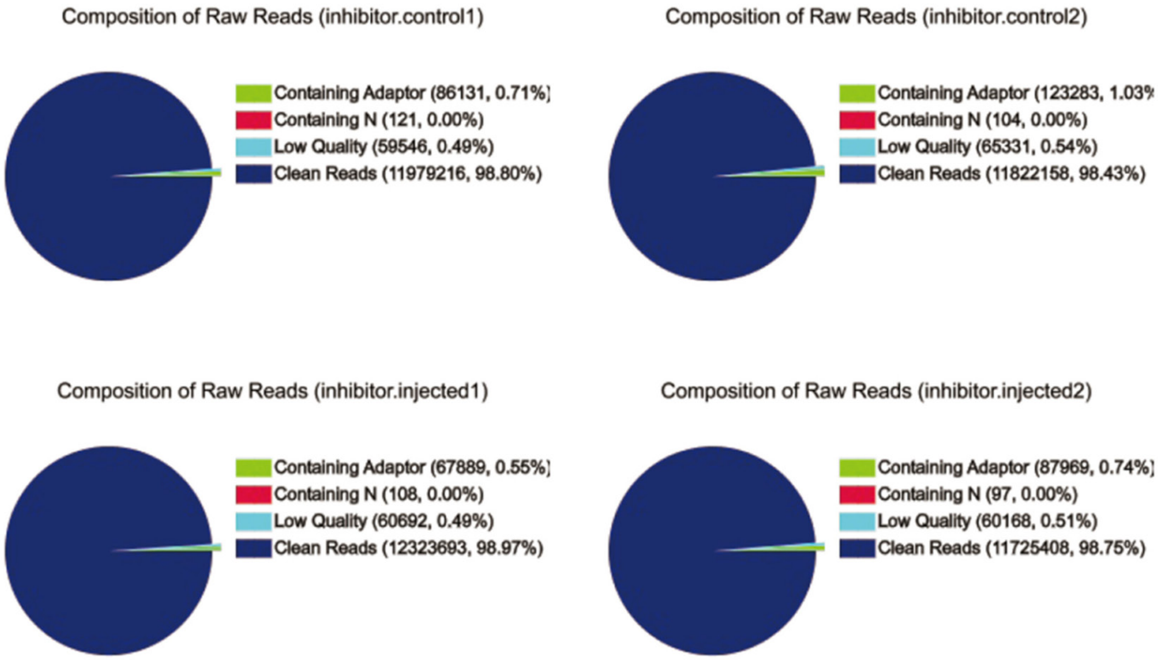

Supplementary Figure S1: Quality assessment of reads.

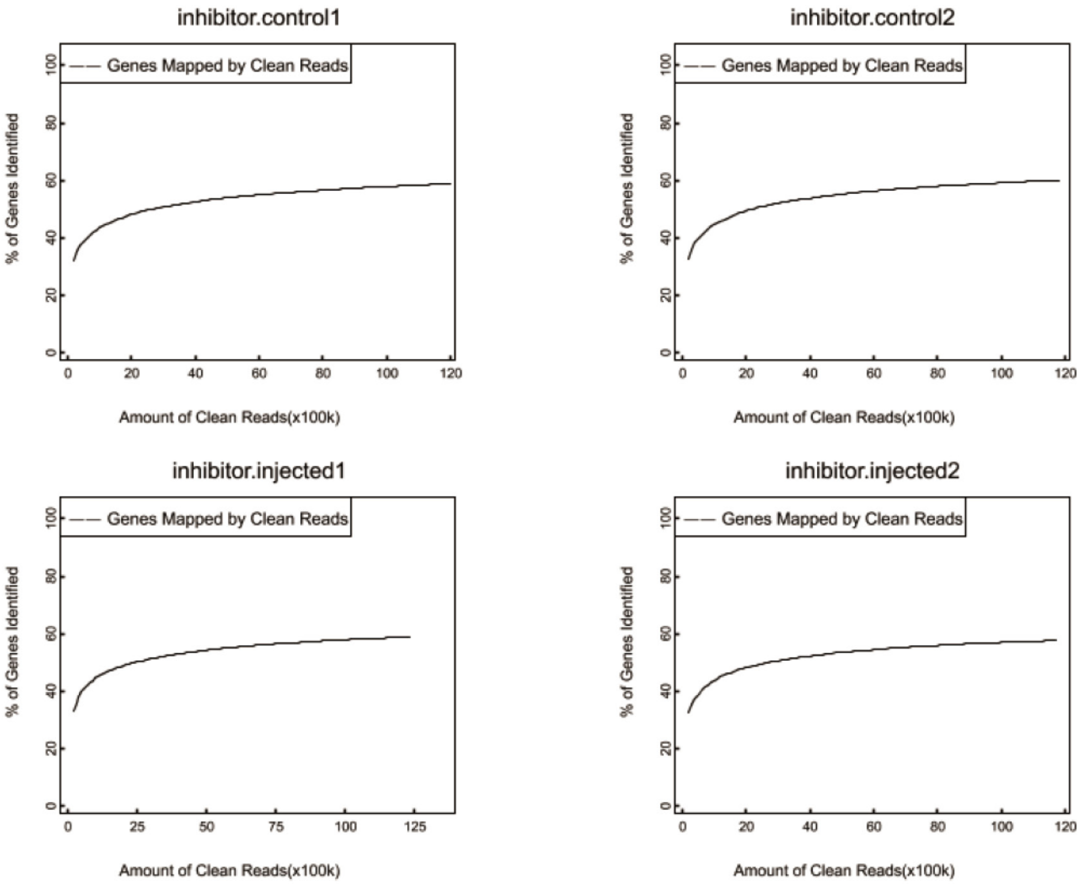

Supplementary Figure S2: Sequencing saturation analysis.

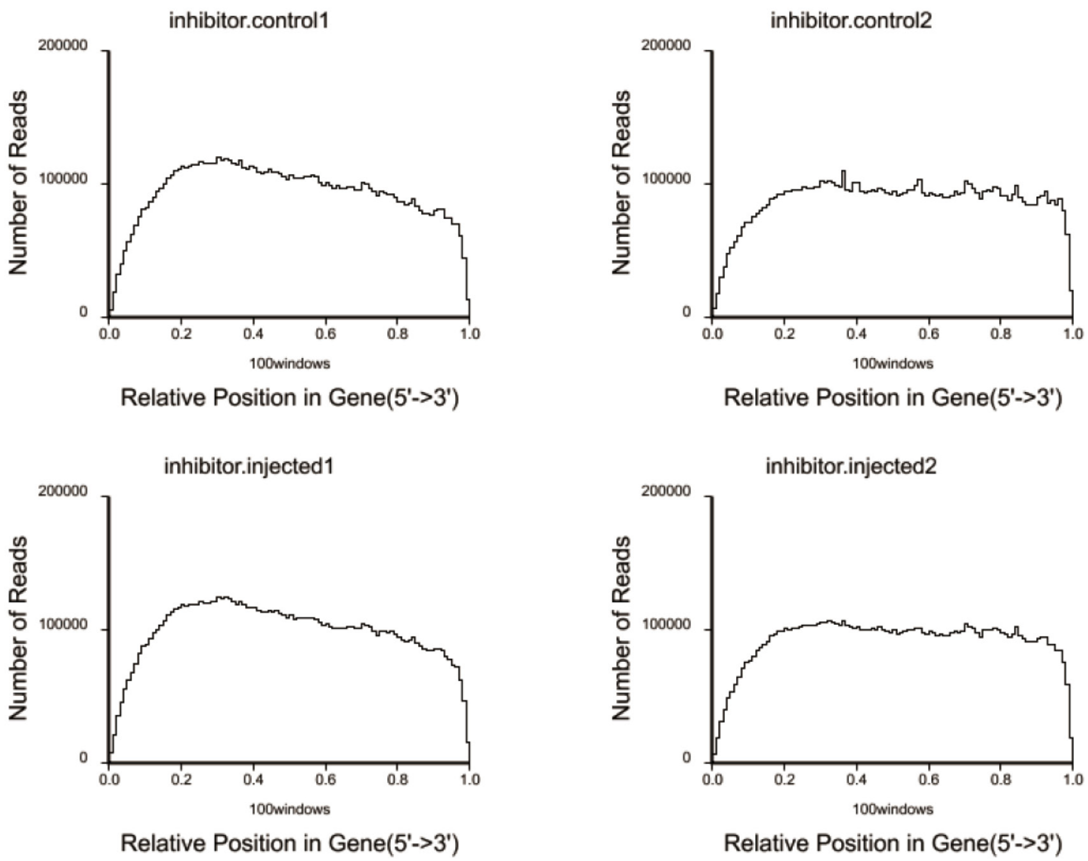

Supplementary Figure S3: Randomness assessment.

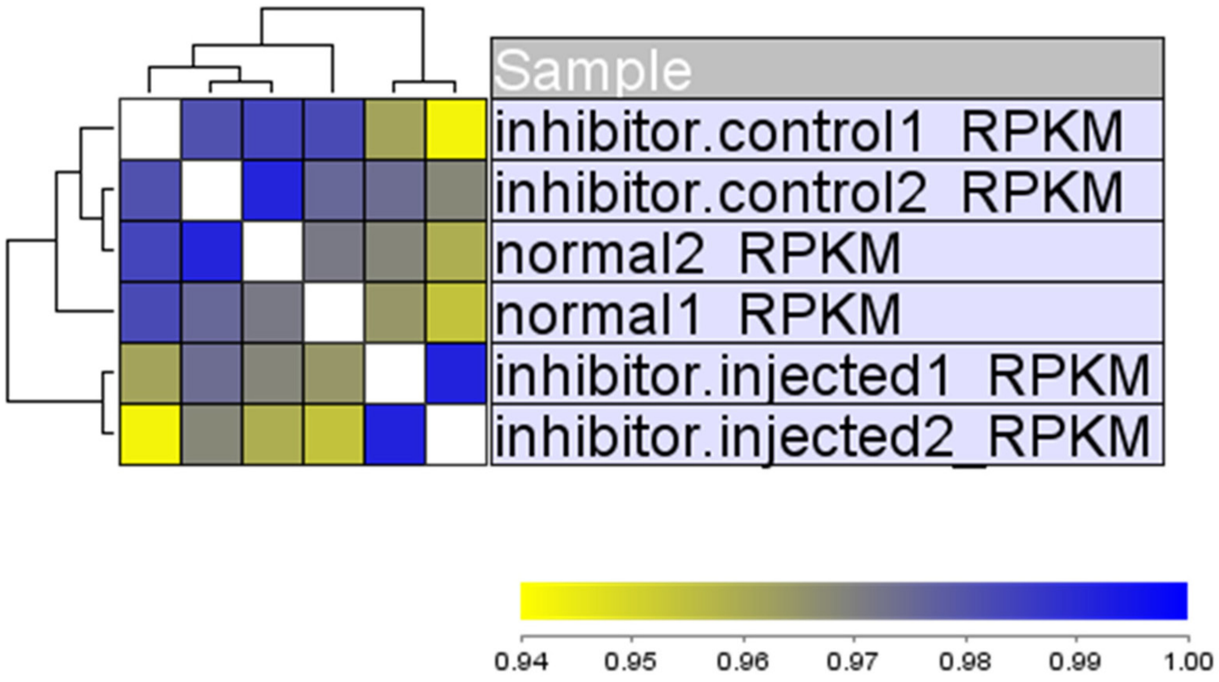

Supplementary Figure S4: Cluster analysis of expression levels in morula-stage embryos following microinjection of miR-29b inhibitor, vehicle control, and without microinjection (normal).

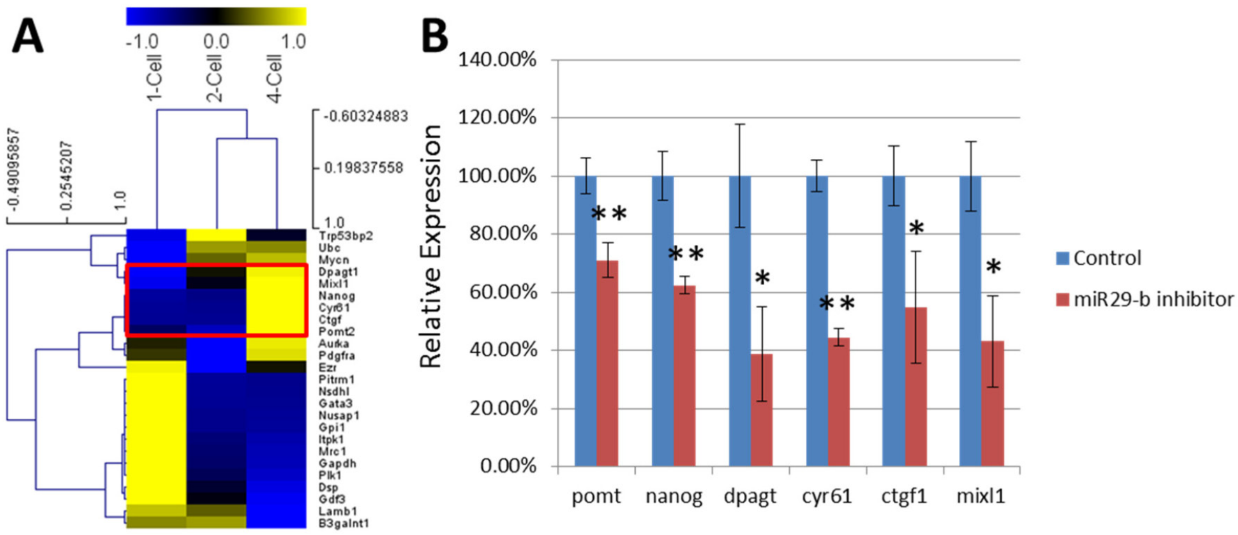

Supplementary Figure S5: Expression patterns of DDEGs with known embryonic lethal phenotypes in null-mutant mouse models. A. Heat map clustering of expression levels of DDEGs at the 1-cell, 2-cell and 4-cell stage. Normalized FPKM value are represented from blue to yellow; **B.** Relative expression of 6 DDEGs in morula-stage embryos that were injected with miR-29b inhibitor (red) or mock control (blue) at the zygote stage. These 6 genes analyzed are normally upregulated during early preimplantation stages (marked by red box in B). *, P-value <0.05; ** P-value <0.01.

Supplementary Table S1: Normalized RPKM of differentially expressed genes (DEGs)

See Supplementary File 1

Supplementary Table S2: Full name of genes

Gene symbol	Full name	Offical Gene Name
Zbtb40	zinc finger and BTB domain containing 40	Zbtb40
Hbp1	high mobility group box transcription factor 1	Hbp1
Ccdc117	coiled-coil domain containing 117	Ccdc117
Ypel2	yippee-like 2 (Drosophila)	Ypel2
Klf4	Kruppel-like factor 4 (gut)	Klf4
Tmed9	transmembrane emp24 protein transport domain containing 9	Tmed9
Oct4	POU domain, class 5, transcription factor 1	Pou5f1
Sox2	SRY (sex determining region Y)-box 2	Sox2
Nanog	Nanog homeobox	Nanog
Pomt2	protein-O-mannosyltransferase 2	Pomt2
Dpagt1	dolichyl-phosphate (UDP-N-acetylglucosamine) acetylglucosaminephosphotransferase 1 (GlcNAc-1-P transferase)	Dpagt1
Mixl1	Mix1 homeobox-like 1 (Xenopus laevis)	Mixl1
Cyr61	cysteine rich protein 61	Cyr61
Ctgf	connective tissue growth factor	Ctgf

Supplementary Table S3: DDEGs that normally undergo upregulation between the 1-cell and the 4-cell stage of mouse preimplantation development

Gene	1-cell	2-cell	4-cell	p_value
Pomt2	0.987526	0.739853	2.03444	0.00345
Nanog	N	4.58485	73.0694	0.00005*
Dpagt1	0.457697	4.04681	6.90076	4.00E-04
Mixl1	0.110126	3.7627	8.71793	0.02335
Cyr61	0.504617	0.953132	25.6374	5.00E-05
Ctgf	0.176113	0.209581	6.42156	0.00345
Zbtb40	3.41001	7.11782	10.0599	5.00E-05
Hbp1	32.4275	71.3712	75.6853	0.00085
Ccdc117	107.971	163.27	225.178	0.00345
Ypel2	54.8243	117.164	226.311	5.00E-05
Klf4	3.82813	17.835	29.0044	5.00E-05
Tmed9	17.1523	79.9583	88.8347	5.00E-05

All P values reflect comparison of 1-cell stage vs 4-cell stage transcript levels per DBTMEE data.
*Nanog was not detectable in the 1-cell stage; the p-value was determined by comparing the 2-cell stage to the 4-cell stage embryo.

Supplementary Table S4: List of primers

Gene Name	Primer Sequence
Pomt2	5'-TTGGGTATGGAAGAAGAGGG-3' (forward)
	5'-CCTACGTGTCCCTCCCTTTA-3' (reverse)
Nanog	5'-GTGCATATACTCTCTCCTTCCC-3' (forward)
	5'-AGCTACCCTCAAACTCCTGGT-3' (reverse)
Dpagt1	5'-GGTGAATTTGATCGGCTCGC-3' (forward)
	5'-GACTCTGGGATCTGCTGCTG-3' (reverse)
Mixl1	5'-GCCCTCACTTGACTGGTTTC-3' (forward)
	5'-GGAAGCTGTTTCCTGAGCTG-3' (reverse)
Cyr61	5'-AGAGGCTTCCTGTCTTTGGC-3' (forward)
	5'-CTCGTGTGGAGATGCCAGTT-3' (reverse)
Ctgf	5'-TTTGCCTGTAACAAGCCAGA-3' (forward)
	5'-TTGAGGCTATCAGTTTAAAATCCC-3' (reverse)
Zbtb40	5'-GCATCCAAGCCTGCTGTACT-3' (forward)
	5'-TAAAGGCACACCCTTCCATC-3' (reverse)
Hbp1	5'-ACCAACTCAGGCTCTCAACAG-3' (forward)
	5'-TGAGACCAAGGTCTTCCAAGC-3' (reverse)
Ccdc117	5'-GGAGGATGATGAGTGTCCAGT-3' (forward)
	5'-CATGCTGGGCATAGAAAGGC-3' (reverse)
Ypel2	5'-TCATTTCCAAGTCCTTCCAGGG-3' (forward)
	5'-AGTCCTGTTAGCAACACCCG-3' (reverse)
Klf4	5'-TGGGGGTTTTGGTTTGAGGT-3' (forward)
	5'-CCAGGTGGCTGCCTCATTA-3' (reverse)
Tmed9	5'-ATCGGAAATTACCGGACGCA-3' (forward)
	5'-CAGGATGACCTTGTCCTCGG-3' (reverse)
18S rRNA	5'-AAACGGCTACCACATCCAAG-3' (forward)
	5'-CCTCCAATGGATCCTCGTTA-3' (reverse)
